# Automatic classification of human facial features based on their appearance

**DOI:** 10.1371/journal.pone.0211314

**Published:** 2019-01-29

**Authors:** Felix Fuentes-Hurtado, Jose A. Diego-Mas, Valery Naranjo, Mariano Alcañiz

**Affiliations:** I3B - Institute for Research and Innovation in Bioengineering, Universitat Politècnica de València, Valencia, Spain; Griffith University, AUSTRALIA

## Abstract

Classification or typology systems used to categorize different human body parts have existed for many years. Nevertheless, there are very few taxonomies of facial features. Ergonomics, forensic anthropology, crime prevention or new human-machine interaction systems and online activities, like e-commerce, e-learning, games, dating or social networks, are fields in which classifications of facial features are useful, for example, to create digital interlocutors that optimize the interactions between human and machines. However, classifying isolated facial features is difficult for human observers. Previous works reported low inter-observer and intra-observer agreement in the evaluation of facial features. This work presents a computer-based procedure to automatically classify facial features based on their global appearance. This procedure deals with the difficulties associated with classifying features using judgements from human observers, and facilitates the development of taxonomies of facial features. Taxonomies obtained through this procedure are presented for eyes, mouths and noses.

## Introduction

Humans have especially developed their perceptual capacity to process faces and to extract information from facial features [1,2]. Our brain has a specialized neural network for processing facial information [3] that allows us to identify people, their gender, age, and race, or even to judge their emotions. Using our behavioral capacity to perceive faces, we make attributions such as personality, intelligence or trustworthiness based on facial appearance [4]. Therefore, faces play a central role in our relationships with other people and in our everyday decisions [5,6].

For centuries, artists and researchers have tried to develop procedures to measure and classify human faces. Anthropometric facial analysis is used in different fields like surgery [7–9], forensic science [10–12], art [13,14], face recognition [15], emotion recognition [16], and facial feature judgments [17–20]. In recent decades, new technologies have opened up ways to automatically evaluate facial features and gestures, and computational methods for the analysis of facial information are now applied to classify faces based on anthropometric or emotional criteria [21].

Classification or typology systems used to categorize different human body parts have existed for many years. In 1940, William Sheldon developed somatotypes to describe the constitution of an individual. Sheldon proposed a classification system in which all possible body types were characterized based on the degree to which they matched these somatotypes [22]. Other taxonomies have been developed for the shape of the body [23,24], hands [25], feet [26] or head [27]. Taxonomies, as a classification system, allow us to use a common terminology to define body part configurations while providing a standardized way to describe them, and are widely used in many fields such as ergonomics and biomechanics [28][29], criminalistics [12], sports [30,31], medicine [32], design or apparel industry [23]. In general, these kind of typology systems are intended for qualitative categorization based on the global appearance of body parts, although, in some cases, a quantitative analysis of some selected features is developed to obtain the classification.

In the case of facial features, taxonomies are useful, for example, in ergonomics, forensic anthropology, crime prevention, human-machine interaction or online activities. E-commerce, e-learning, games, dating or social networks, are fields in which classifications of facial features are needed. In these activities it is common to use human digital representations that symbolize the user’s presence or that act as a virtual interlocutor [33]. The importance of communicative behaviors of avatars in new interaction systems [34–37] has led to an increasing interest in creating realistic avatars able to convey appropriate sensations to users. In this context, it is common to synthesize faces and facial expressions combining facial features [38–41].

Several taxonomies of facial features can be found in the literature. For example, Vanezis’s atlas [42] classifies 23 facial features, the Disaster Victim Identification Form (DVI) by Interpol categorizes 6, and the DVM database [43,44] 45 facial traits. In [45] different shapes of the human nose are classified into 14 groups based on the analysis of 1,793 pictures of noses. A similar approach was used to classify human chins [46]. In these works, a big set of photographs were analyzed and classified based on the similarity of the features.

This approach, while intuitively logical, has several problems not only in the development of taxonomies, but also in its subsequent use. The classification of facial features is obtained from the opinion of a limited group of human observers. Classic behavioral work has shown that the human brain integrates facial features into a gestalt whole when it processes facial information (holistic face processing) [47], decreasing our ability for processing individual features or parts of faces [48]. This part-whole effect makes it difficult, for example, to recognize familiar faces from isolated features [49–51]. Moreover, individual differences exist in face recognition ability [52], and some matters, like the race of the face, influence the performance in processing features and the configuration of facial information [53,54]. This is reflected in low inter-observer and intra-observer agreement in the evaluation of facial features [12]. Finally, apart from the difficulties of processing parts of faces, creating these kinds of taxonomy implies classifying a very big set of elements (the number of possible different features) in an undefined number of groups, and this kind of task easily overcomes our capacities for information processing [55,56]. To deal with these problems, we propose a new procedure to develop taxonomies of facial features based on their appearance, using computational methods for automatically classifying features.

Recently, analysis of facial images has become a major research topic, and new computational methods for analysis of facial information have been developed. A comparison of these techniques shows two different approaches to deal with facial information [19]. The first one (structural approach) automatically encodes the geometry of faces using several significant points and relationships between them, carrying out a metric or morphological assessment of facial features [57]. Examples of these kinds of techniques are those based on SIFT feature descriptors [58,59], point distribution models [60,61] or local binary patterns [62–64]. On the other hand, the holistic approach uses appearance-based representations, considering all available information and encompassing the global nature of the faces. Holistic techniques include, for example, fisherfaces [65] or eigenfaces [66]. Some work in facial features characterization has been done mixing structural and holistic techniques [67].

Classification methods of facial features are needed in order to develop taxonomies. Research using computational methods is usually focused on the characterization of complete faces. However, less efforts have been made in classification of facial features based on their appearance. In this work, we use an appearance-based method to obtain a relatively low-dimensional vector of characteristics for facial features. On this basis, large sets of three facial features (noses, mouths, and eyes) of varying ethnicity (Asian, Black, Latino, and White) were characterized. Using this characterization, the features were clustered obtaining new taxonomies for each ethnic group. The procedure followed avoids the problems related to human limitations in classifying facial features. On the one hand, the characterization and clustering of the features were not based on human judgements. On the other hand, classifying new features in one of the groups of the taxonomies can be done in an automatized way. Finally, the procedure was tested comparing human opinions with automatically generated groups of facial features.

The next section shows the preliminary process of treatment of images to obtain large sets of facial features from photographs of complete faces. Afterwards, we used eigenfaces in order to characterize large sets of photographs of three facial features (noses, mouths, and eyes). This holistic technique seems to be more consistent and reliable for categorizations than those that imply subjective judgements [19]. The clustering process used to group features is also shown. Next, we present the classifications obtained and the agreement between human judgements and these automatically generated taxonomies. Finally, the results are discussed and conclusions are shown.

## Whole face image preprocessing

Our first objective was to obtain a large database of facial features of different ethnic groups with a neutral expression. Many real face databases are accessible for research purposes [68], however, to the best of our knowledge, there are not large public databases of real facial features available. Therefore, we developed an algorithm to process images from a whole face database and to extract images of the facial features.

The available datasets differ in the size and resolution of the images, the pose and orientations of the faces, the uniformity of the background, the illumination, and other important aspects. After reviewing several well-known databases, we selected the Chicago Face Database [69] to extract images of the facial features. After its second revision, this database contains high-resolution standardized images of real faces of Asian, Black, Latino, and White males and females with several expressions (including neutral). 290 images of males with neutral expression (93 Black, 52 Asian, 52 Latino, and 93 White) were used to create four subsets of face images (one per ethnic group).

**Fig 1:**
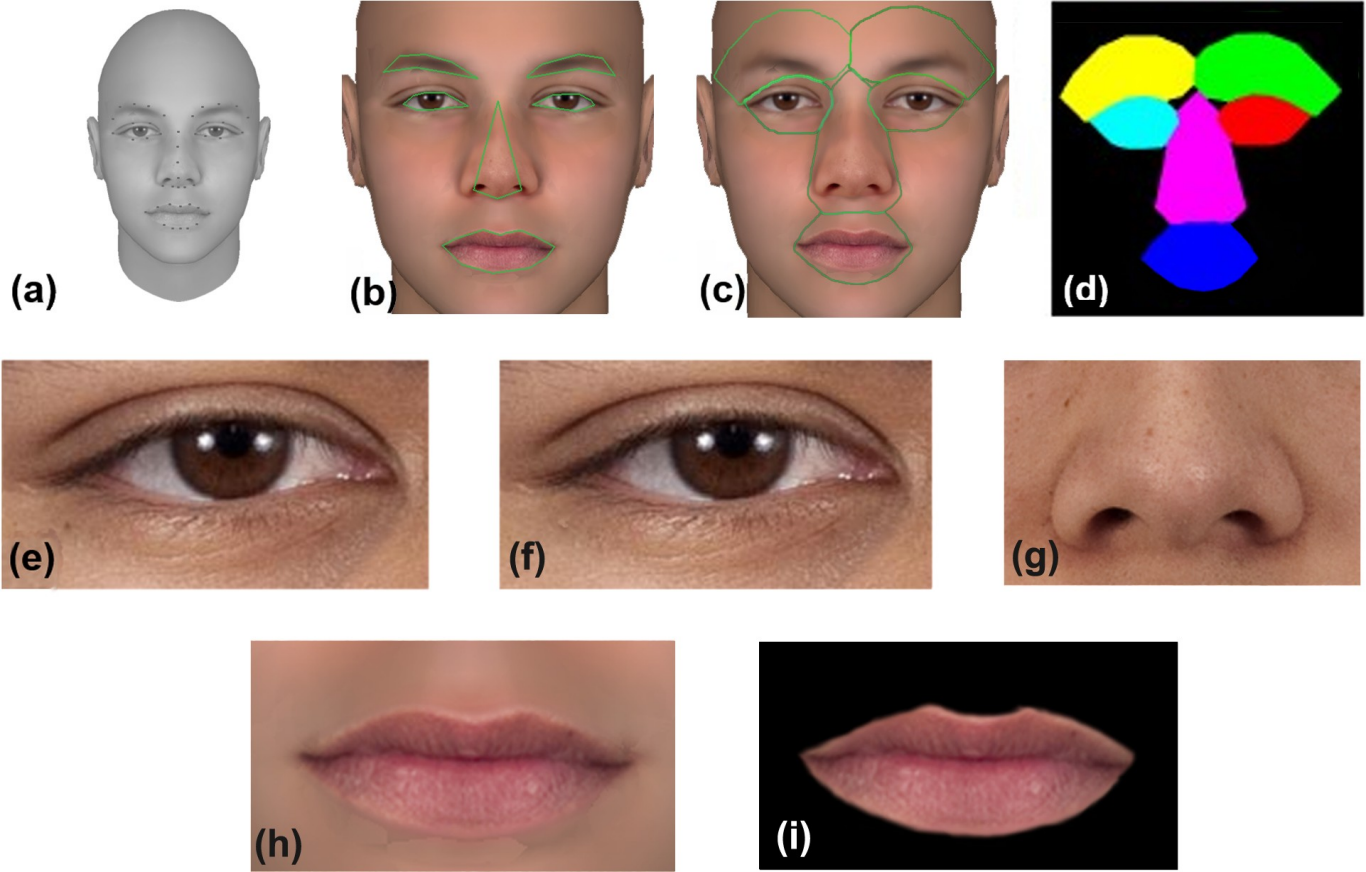
Masks creation for feature extraction. (a) Landmarks distribution. (b) Mask created from landmarks. (c) Thickened mask. (d) Independent masks for each feature. (e) Right eye. (f) Mirrored left eye. (g) Extracted nose. (h) Original mouth. (i) Shaved mouth.

**Fig 2:**
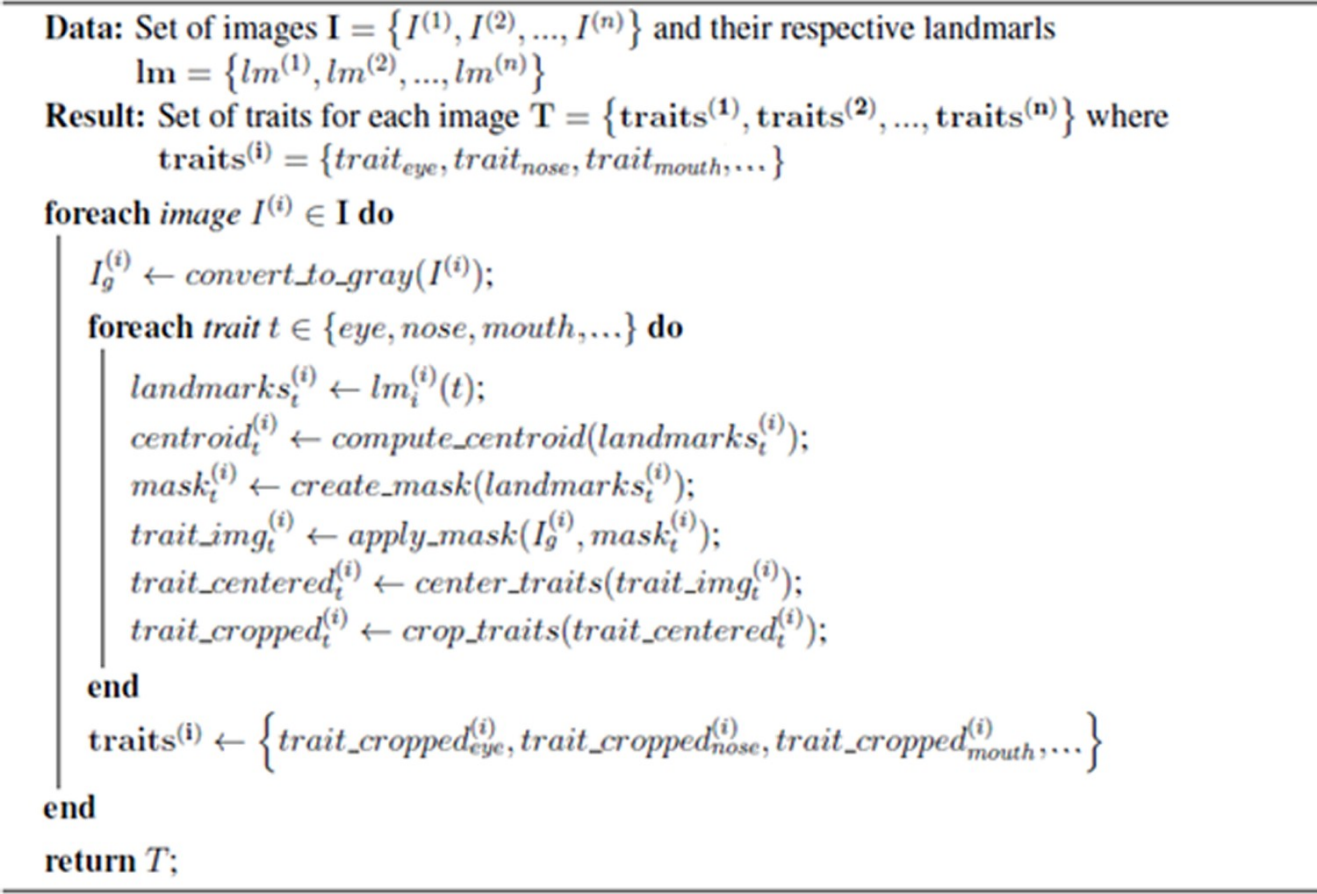
Pseudo-code of the algorithm to extract the features from the whole face photographs.

The input of the algorithm for facial feature extraction were all the RGB full-face photographs. Initially, the images were converted to gray-scale. Next, the facial landmarks of each feature (eyes, mouth, and nose) were detected and each feature separately extracted in images of same size for each feature. To achieve this, the CHEHRA facial key-point detector [70] was used. The outcome was a set of 49 landmarks distributed as shown in Fig 1 (A). Based on these landmarks, a mask for each feature was automatically created (Fig 1 (D)). Using these masks, the part of the image corresponding to each facial feature was separated. The procedure to extract the features from the whole face photographs is detailed as a pseudo-code algorithm in Fig 2.

Once the features of the faces are available in independent files, each family of them (i.e. eyes, noses, and mouths) goes through a set of different operations. The first process performed over the features’ images was an alignment operation. For every feature, a polygon was formed using the previously acquired landmarks and its centroid was computed. Then, all the features were aligned using the previously calculated centroids as reference. After that, the size of the bounding box of the polygon created by the landmarks was computed, and a mask was created to crop all features to the size of the biggest bounding box. By performing this, the cropping rectangle fits the feature itself in the most tight-fitting way possible, discarding as much skin as possible to avoid noise in the clustering step. This procedure was performed for each kind of feature, obtaining the results shown in Fig 1.

Before saving them as independent files, eyes and mouths required a special treatment. On the one hand, two eyes were obtained from each face. Except in very particular cases, one person's eyes are highly symmetrical and both must be classified in the same group when using appearance as criterion to cluster the eyes. Therefore, they can be used as an indicator of the correctness of a clustering process, and we decided to use both eyes of each face. To homogenize the appearance of the eyes, left eyes images were mirrored horizontally before saving them (Fig 1 (F)). On the other hand, is common the presence of hair around the mouth of men. In our first tests we detected that the presence of hair greatly affected the process of grouping the mouths, therefore, we decided to remove the surroundings of the original mouth (Fig 1 (H)), obtaining a “shaved” mouth (Fig 1 (I)).

The procedure followed to “shave” the mouths was as follows: first, the outer landmarks of the mouth were selected to form a polygon. Then, this polygon was enlarged by 5 pixels in every direction to ensure all the mouth was taken inside the mask. Finally, a Gaussian Blur Filter (sigma = 2) [71] was applied to the mask in order to smooth the transition between the skin and the black background of the image (Fig 1 (I)).

### Proposed procedure for automatic classification of facial features

**Fig 3:**
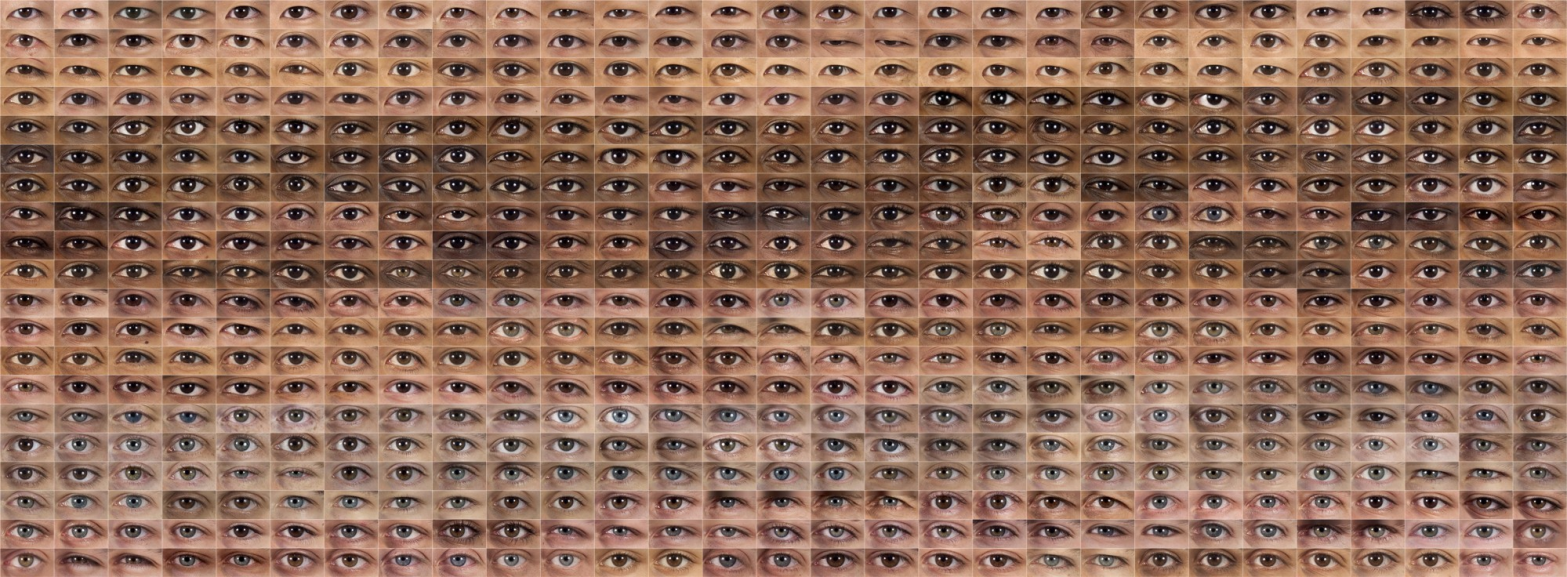
Dataset of 580 images of eyes obtained using the extraction algorithm.

At this stage, sets of 290 noses, 290 “shaved” mouths, and 580 eyes (Fig 3) were available. Several techniques could be used for data reduction and feature extraction, and to group the facial features. Holistic models based on principal component analysis, like fisherfaces and eigenfaces, have proved their suitability in face detection, recognition and face judgements, and are currently used in applications in which process speed and resource consumption are critical [72–76]. On the other hand, artificial neural networks, support vector machines and deep learning methods [77,78] are currently able to jointly optimize feature extraction and clustering, yielding better results than sequentially applying them [79].

Our objective was to develop taxonomies of human facial features in a simple and automatized way; therefore, our criteria to select the most suitable techniques were efficiency and simplicity. We tested different combinations of procedures like eigenfaces, fisherfaces and autoencoders [80] for feature extraction; hybrid PCA/multilayer perceptron networks and convolutional neural networks for feature extraction; as well as K-means, G-means [81] and DBScan [82] for clustering. Our initial tests found that the results obtained sequentially using eigenfaces and K-means were almost equal to those obtained using more complex processes. As our criteria to select the most suitable techniques for our procedure were efficiency and simplicity, we finally selected eigenfaces and K-means. Both are well known techniques, easy to implement, fast and efficient and have only a few parameters to tune. As a drawback, eigenfaces is a global appearance method that is less robust to face misalignment and background variations than other procedures. However, in the previous image preprocessing stage, the facial features were aligned and the background removed.

Therefore, eigenfaces were used in order to characterize each feature of each dataset (we maintain the term eigenfaces although we used it over facial features). Finally, the K-Means clustering algorithm [51] was used to clusterize the features using their eigenvalues as characteristics.

#### Using eigenfaces on features

The eigenfaces approach is a method to efficiently represent pictures of faces by a relatively low-dimensional vector. A principal component analysis can be used on an ensemble of face images to form a set of basis features [83]. These basis images, known as eigenpictures, can be linearly combined to reconstruct images in the original set.

In mathematical terms, the eigenfaces method aims to find the principal components of the distribution of faces, or the eigenvectors of the covariance matrix of the set of face images, treating each image as a vector in a very high dimensional space. These eigenvectors (or eigenfaces) can be thought of as a set of features that together characterize the variation between images, and are ordered accounting for the explained variance. Each individual face can be represented exactly in terms of a linear combination of the eigenfaces, or using the "best" eigenfaces (those that explain the largest variances, and therefore account for the most variation within the set of images). The best M eigenfaces span an M-dimensional subspace of all possible images. Using this procedure over each set of features it was possible to characterize each feature by a set of M eigenvalues, reducing the quantity of information used to describe the features. This holistic approach was selected to characterize the features because the objective was to classify them based on their global appearance more than on their geometrical characteristics (structural approach). This procedure allow us to consider the global appearance of faces while summarizing the central information to characterize them.

The Eigenfaces method was applied over each subset of facial features. To facilitate the subsequent clustering process, the same number of eigenfaces (45) for each subset was selected bearing in mind that the explained variances were about 85% or higher in all cases (Table 1).

**Table 1:** Percentages of variance explained by 45 eigenfaces for each dataset.

	Ethnic group			
Feature	Asian	Black	Latino	White
**Eyes**	91.15	84.98	88.61	86.22
**Noses**	98.55	93.49	98.19	91.36
**Mouths**	98.88	93.69	99.14	95.26

**Fig 4:**
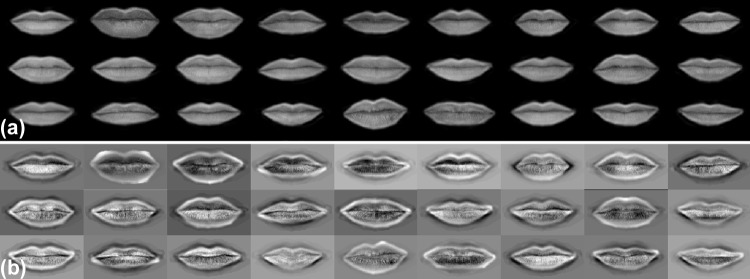
Original and reconstructed mouths before de-normalization using 45 eigenfaces. (a) Original mouths. (b) Reconstructed mouths.

At this stage, the appearance of each feature could be characterized using 45 real values (eigenvalues). As an example of the information of the features that was captured using eigenfaces, Fig 4 shows a reduced set of original mouths (a), and the same set of mouths reconstructed using 45 eigenvalues before de-normalization (b).

#### Clustering the facial features

The K-Means clustering algorithm [51] was selected to cluster the features using their eigenvalues as characteristics. A drawback of using this method is that the number of clusters (K) must be predefined. The approach used to deal with this problem was to perform several K-Means executions varying K, and to calculate the Dunn’s Index [53] for each set of clusters. The Dunn’s Index measures the compactness and separation of the clusters obtained for each K. A higher Dunn’s Index points to a small intra-cluster variance and a high inter-cluster distance, i.e. the features included in each cluster are more similar to each other, and more different from the features belonging to other clusters. Therefore, the number of clusters for each feature was selected as the K that maximized the Dunn’s Index.

## Results

Four subsets (Asian, Black, Latino, and White) of three facial features (eyes, noses, and mouths) previously obtained were grouped according to their appearance, measured through 45 eigenvalues, using the K-Means clustering algorithm. In order to determine the most suitable number of clusters, several runs of the algorithm were performed increasing the K from 5 to 30, and the Dunn’s Index for each obtained set of clusters was calculated. The results of iterative clustering algorithms like K-Means can vary depending on the initialization, which consists of selecting random initial positions for the clusters. That could yield different results in each execution; therefore, a round of 10 K-Means runs for each K were performed to check the coherence of the results throughout executions. The experiment was implemented using Matlab R2016a on an Intel(R) Core(TM) i7-4770S at 3.10GHz processor PC with 16 GB of RAM.

**Fig 5:**
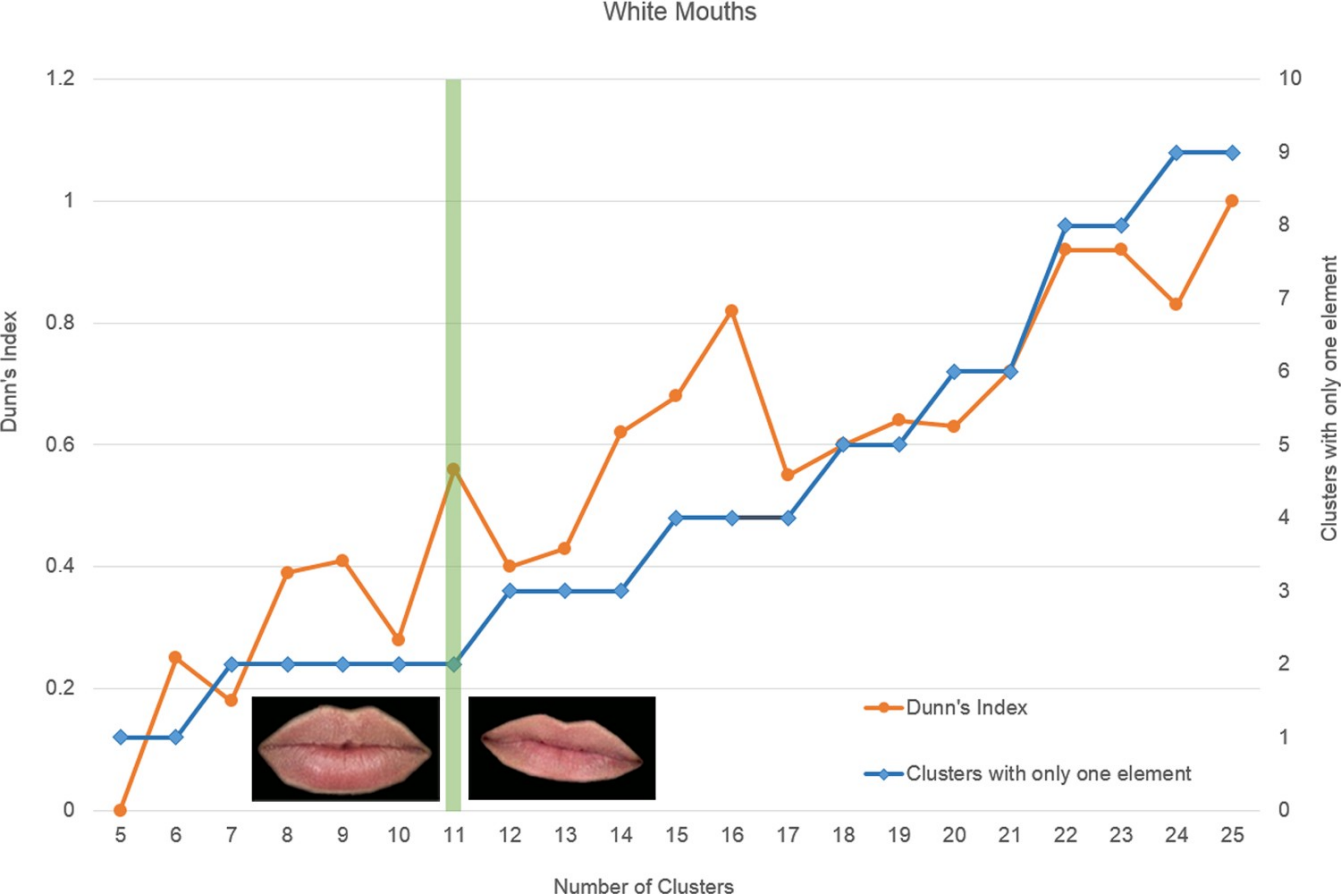
Dunn’s Index and clusters with a single element per number of clusters for white mouths.

As an example of how the number of clusters was selected for each subset, Fig 5 shows the Dunn’s Index obtained for each K for the case of white mouths, and the number of clusters with a single element (SEC) per total number of clusters. As can be seen, high Dunn’s Index values tend to be associated with high values of K, however, the number of SECs also increases with K. SECs were usually formed by features that have had some problem in the previous automatic preprocessing of the image (centering, cropping or resizing), and can be considered outliers. For these reasons, the optimal number of clusters was selected as the K that produced higher Dunn’s Index and two or less SECs. After that, SECs were reviewed and eliminated if their elements were considered outliers. For the mouths and the noses, SECs were those formed by only one mouth or one nose. For the eyes, SECs were those formed by less than a pair of eyes. In this way, clusters containing only one individual eye, or containing only the two eyes of the same person, or two eyes of different people, were all considered SECs.

In the case of the white mouths, the highest Dunn’s Index was obtained for K = 11 (being SEC number ≤2). Fig 5 shows the image of the mouths belonging to the two SECs. One of them was considered an outlier because its size was very large with respect to the size of the image, and the other one because it was rotated with respect to the horizontal axis. Therefore, these clusters were not considered and only 9 clusters were used for this subset.

**Fig 6:**
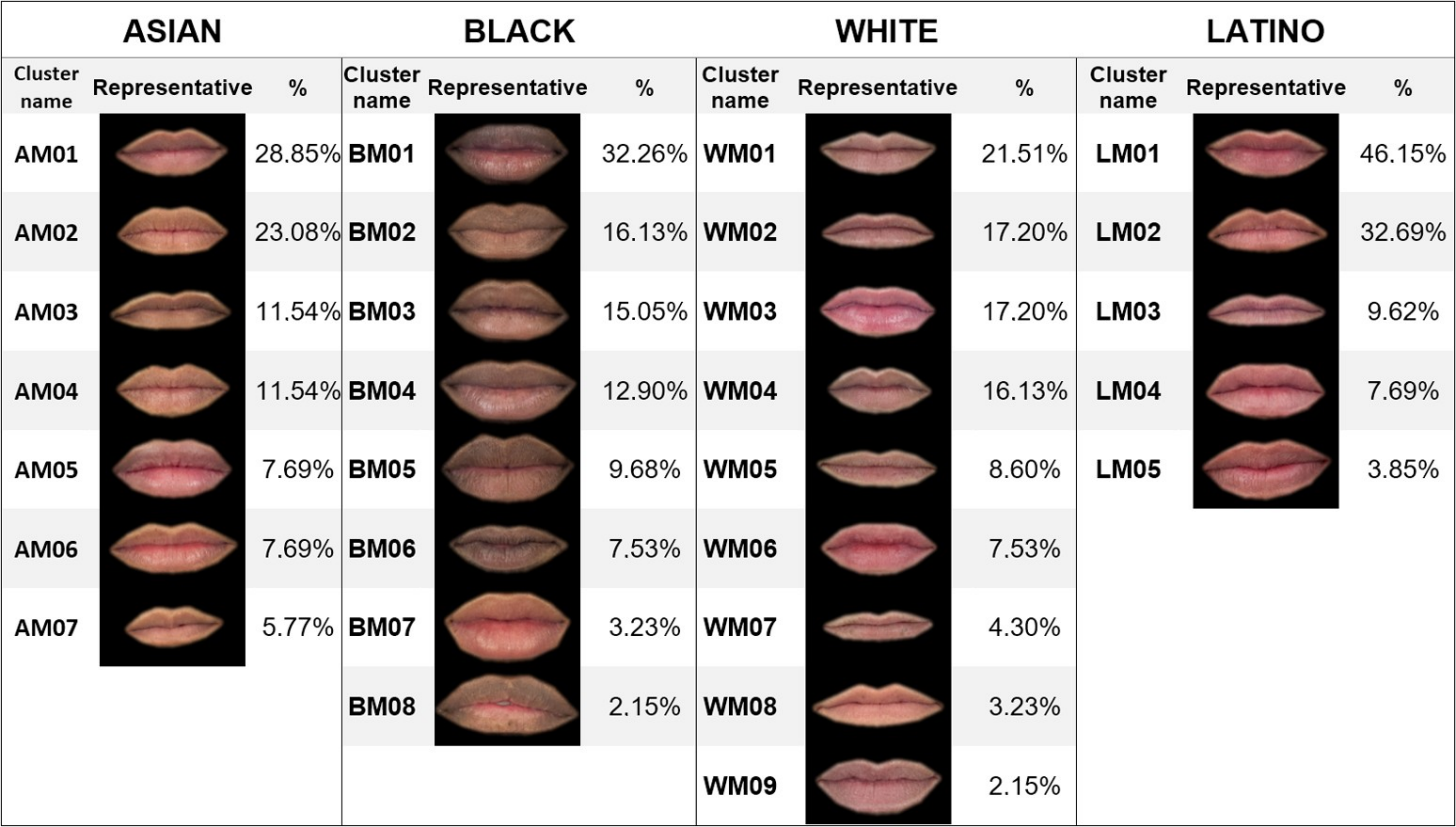
Taxonomy of mouths. The name, the representative feature, and the membership percentage of each cluster are shown for each ethnic group.

**Fig 7:**
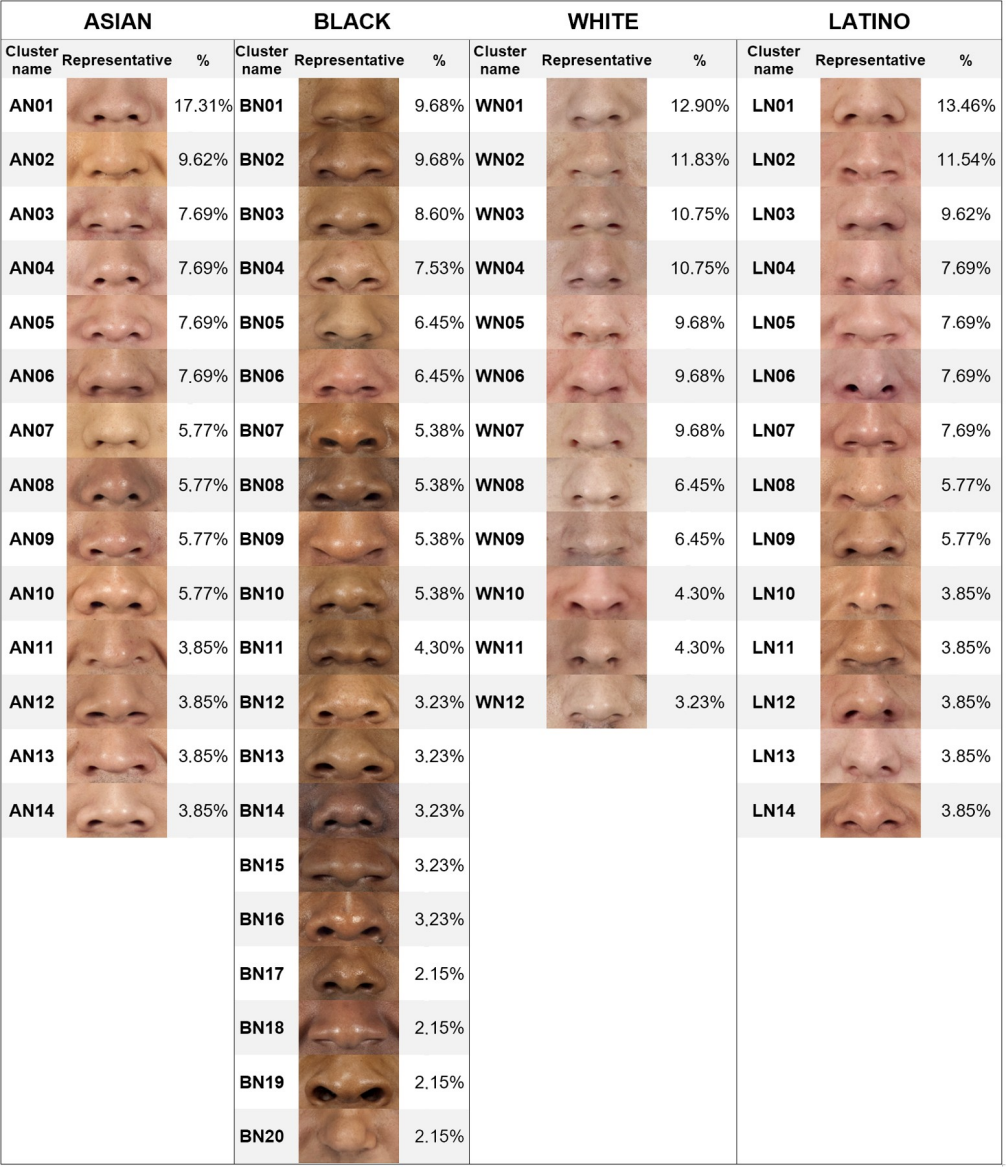
Taxonomy of noses. The name, the representative feature, and the membership percentage of each cluster are shown for each ethnic group.

**Fig 8:**
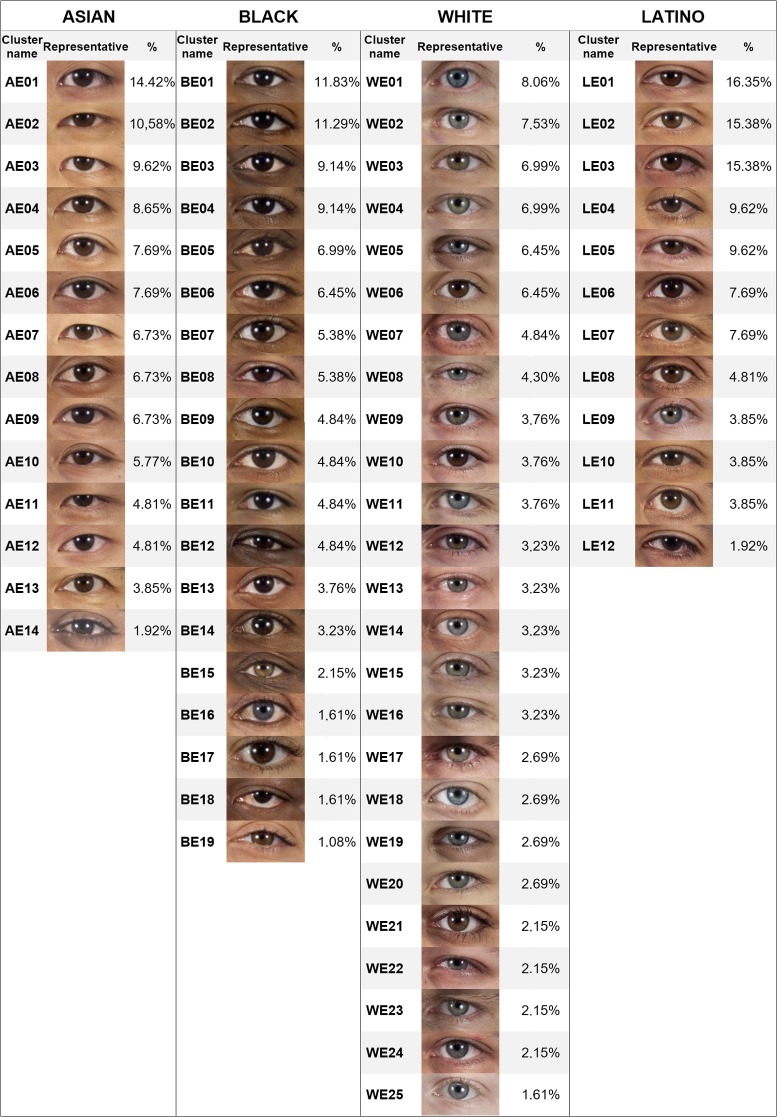
Taxonomy of eyes. The name, the representative feature, and the membership percentage of each cluster are shown for each ethnic group.

**Fig 9:**
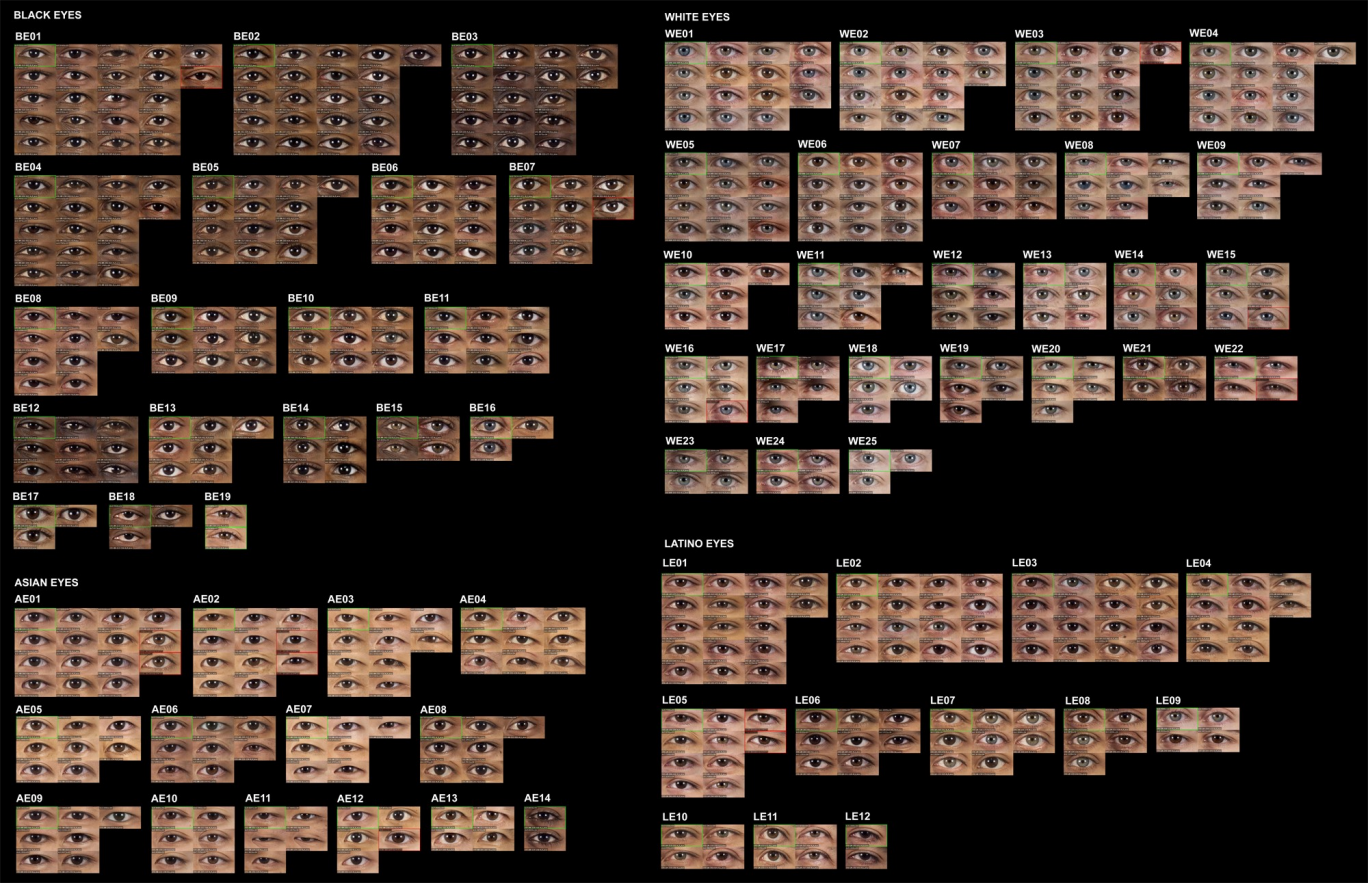
Clusters of Black, White, Latino and Asian eyes.

**Fig 10:**
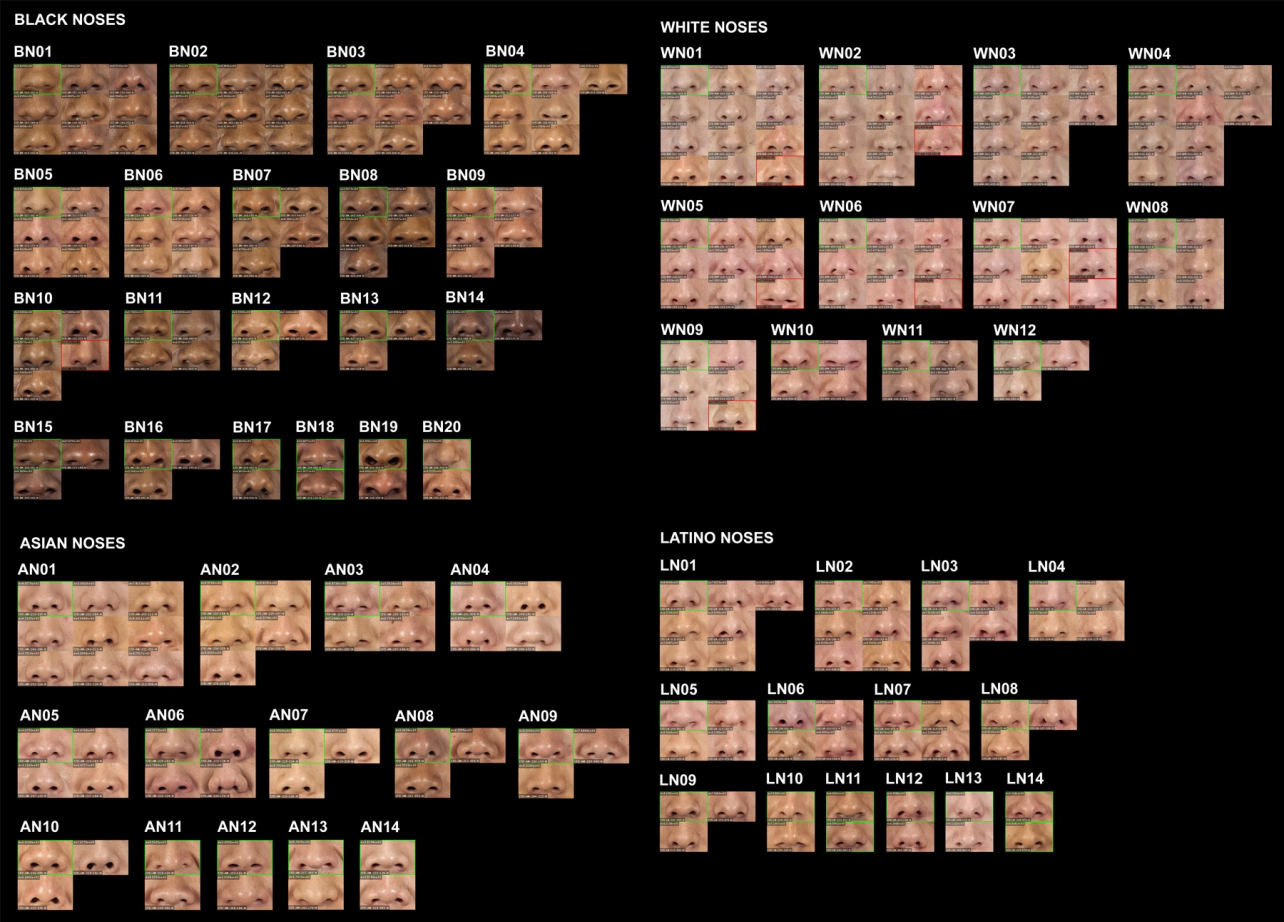
Clusters of Black, White, Latino and Asian noses.

**Fig 11:**
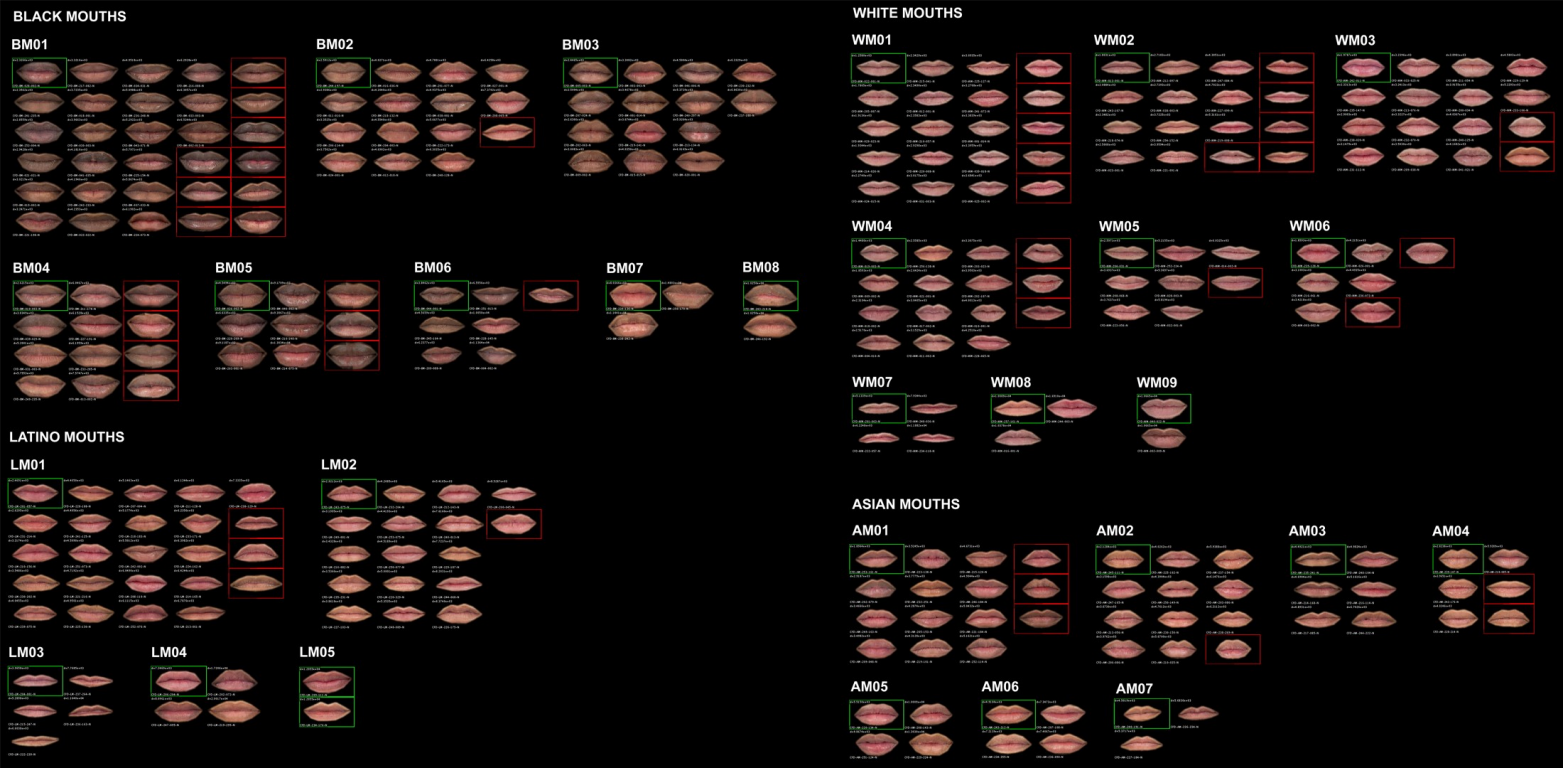
Clusters of Black, White, Latino and Asian mouths.

The same procedure was performed for each subset. Table 2 shows the number of clusters finally obtained for each feature and ethnic group. The percentage of elements in each cluster over the total number of elements in each subset was calculated, and the clusters were sorted from highest to lowest percentage. To identify the clusters, a code composed of four digits was assigned to each one. The first digit was A (Asian), B (Black), L (Latino) or W (White). The second was M (mouth), N (nose) or E (eye). The two last digits were the order of the cluster in its subset. For example, cluster AM01 was the most populated cluster of mouths for Asian ethnicity, and WN12 the least populated cluster of noses for White people. Finally, the closest features to the center of their clusters were selected as representatives of their groups. Figs 6–8 show the obtained classification for each feature, and Figs 9–11 present the complete set of clusters for eyes, noses and mouths. The images of all the clusters are available for download on https://www.ergonautas.upv.es/lab/facial_features/clusters/).

**Table 2:** Number of clusters for each feature for each ethnic group.

	Ethnic group			
Feature	Asian	Black	Latino	White
**Eyes**	14	19	12	25
**Noses**	14	20	14	12
**Mouths**	7	8	5	9

**Fig 12:**
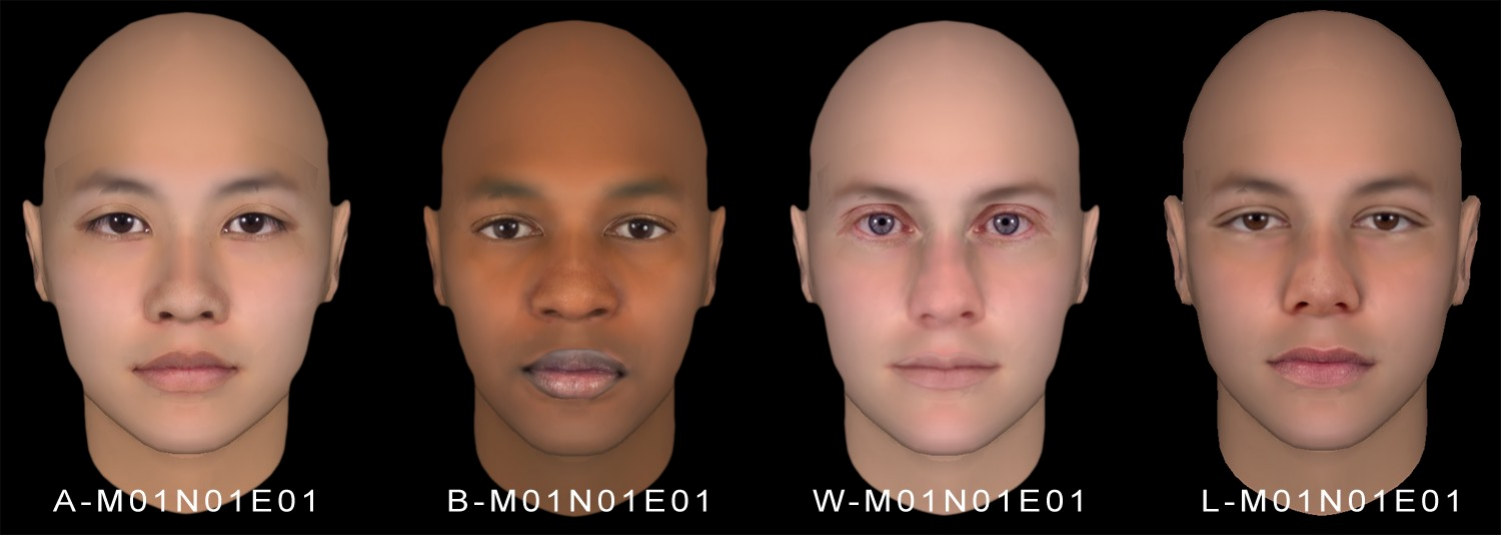
Codification of faces composed using the representative facial features of the most populated clusters for each ethnic group.

The codification employed for the features was expanded to classify whole faces considering their features. In this case, the first digit indicates the ethnic group of the face, i.e. A (Asian), B (Black), L (Latino) or W (White). After a hyphen, three groups of three digits express the mouth, nose, and eyes cluster. As an example, in Fig 12 four faces were composed using the representative features of the most populated clusters for each ethnic group (A-M01N01E01, B-M01N01E01, W-M01N01E01, and L-M01N01E01). The representative features of the most populated clusters are illustrative of the most typical features in the face database employed to obtain this taxonomy.

### Validation of the procedure

This work proposes an automatic procedure to classify features based on their appearance. This procedure was used to group features of faces extracted from the Chicago Face Database. The intuitively logical approach to validate the procedure is to compare the obtained taxonomies with those generated by human evaluators. However, as aforementioned (Section 1), this last approach has important drawbacks. Classifying a big set of features in an undefined number of groups is a hard task considering human capabilities for information processing [55,56]. On the other hand, some important problems of using this approach are the part-whole effect [48], that decreases human ability for processing individual features, and the influence of the race of the face on the performance in processing facial information [53,54]. Previous works have reported low inter-observer and intra-observer agreement in the evaluation of facial features [12]; therefore, a different approach must be used to validate the proposed procedure.

**Fig 13:**
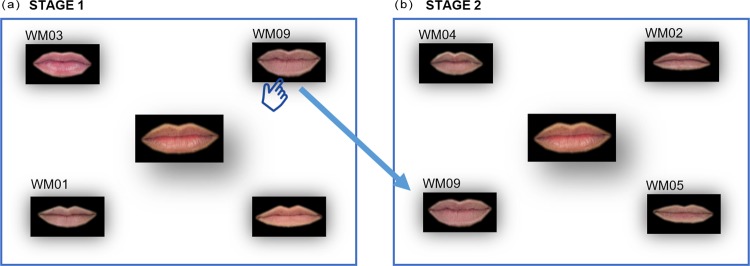
Stages 1 and 2 of the survey procedure.

Instead of comparing the obtained taxonomies with those generated by humans, we measured the agreement of human evaluators with the proposed taxonomies. The main objectives were: to reduce the number of features presented simultaneously to the human evaluators in order to make a decision, and to simplify the decision that must be made. To do this, a survey composed of several stages was developed. Initially, the image of one feature was selected from the entire dataset in a random way (target feature). Four different representative features were randomly selected (representative features are those designated as representatives of their groups in the obtained taxonomy). In the first stage of the survey the five features were presented to the evaluator in a web form (Fig 13 (A)). The target feature was in the center of the form, and the four representative features were at the corners. The evaluator was asked to select the representative feature most similar to the target feature clicking it using the mouse. The request presented to the participants was: “Please select the eye/nose/mouth most similar to the one shown in the center of the screen”. Once the participant made the decision, the selected representative feature passed to the second stage in which a new form was composed as in Fig 13 (B). The target feature was in the center again, and the selected representative feature was at a corner of the form. Three new different representative features were randomly selected and situated in the three remaining corners. This process was repeated until each representative feature was shown at least once. The cluster of the representative feature selected in the last stage was considered to be the result of the survey (i.e. the cluster to which the target feature belongs according to the opinion of the respondent). Using this procedure, the decision-making process was simplified because the number of simultaneous alternatives was reduced to four. As a drawback, the probability of one representative feature to be finally selected depends slightly on the stage in which it is shown.

21 white males and 11 white females aged between 25 and 46 years old participated in three surveys (mouths, eyes and noses). The Comité de Ética en la Investigación (Institutional Review Board of the Universidad Politécnica de Valencia) reviewed and approved these studies. Participants were recruited from May to July 2017 through internal media coverage of the study in the university. Participants gave written informed consent according to the procedures of the Universidad Politécnica de Valencia. The surveys were carried out at the Instituto de Investigación e Innovación en Bioingeniería in Valencia, Spain. In each survey, 200 target features were selected at random from each white features dataset, excluding the representative features. The target features were presented in the survey web form, and the cluster of the representative feature finally selected by the evaluators was registered.

Table 3 shows the results of the survey. The first column of this table presents the cluster finally selected. In this column, Expected refers to the cluster in which the target feature was grouped by the automatic procedure. 82 target mouths, 62 target eyes and 93 target noses were classified in the expected cluster. The distance between clusters can be measured through the eigenvalues of their representative features; therefore, it is possible to determine the distance from the expected cluster to each of the other clusters. The closer two clusters are, the more similar are the features they contain. In Table 3, 1st closest is the cluster nearest to the expected cluster, 2nd closest is the second cluster nearest to the expected cluster and so on. The number, the percentage and the cumulative percentage of features classified in each cluster are shown. The percentages of features classified in the expected cluster or in the three clusters closest to it were 75.5% for mouths, 73.0% for eyes and 81.0% for noses.

**Table 3:** Results of the validation survey.

	Mouths			Eyes			Noses		
Selected cluster	Nº	%	Cum %	Nº	%	Cum %	Nº	%	Cum %
**Expected**	82	41.0%	41.0%	62	31.0%	31.0%	93	46.5%	46.5%
**1st closest**	24	12.0%	53.0%	38	19.0%	50.0%	36	18.0%	64.5%
**2nd closest**	23	11.5%	64.5%	27	13.5%	63.5%	21	10.5%	75.0%
**3rd closest**	22	11.0%	75.5%	19	9.5%	73.0%	12	6.0%	81.0%
**4th closest**	17	8.5%	84.0%	12	6.0%	79.0%	8	4.0%	85.0%
**5th closest**	14	7.0%	91.0%	8	4.0%	83.0%	14	7.0%	92.0%
**6th closest**	6	3.0%	94.0%	5	2.5%	85.5%	3	1.5%	93.5%
**7th closest**	6	3.0%	97.0%	9	4.5%	90.0%	5	2.5%	96.0%
**8th closest**	6	3.0%	100.0%	4	2.0%	92.0%	6	3.0%	99.0%
**9th closest**	-	-	-	1	0.5%	92.5%	1	0.5%	99.5%
**10th closest**	-	-	-	5	2.5%	95.0%	0	0.0%	99.5%
**11th closest**	-	-	-	3	1.5%	96.5%	1	0.5%	100.0%
**12th closest**	-	-	-	1	0.5%	97.0%	-	-	-
**13th closest**	-	-	-	0	0.0%	97.0%	-	-	-
**14th closest**	-	-	-	2	1.0%	98.0%	-	-	-
**15th closest**	-	-	-	0	0.0%	98.0%	-	-	-
**16th closest**	-	-	-	1	0.5%	98.5%	-	-	-
**17th closest**	-	-	-	0	0.0%	98.5%	-	-	-
**18th closest**	-	-	-	1	0.5%	99.0%	-	-	-
**19th closest**	-	-	-	0	0.0%	99.0%	-	-	-
**20th closest**	-	-	-	0	0.0%	99.0%	-	-	-
**21th closest**	-	-	-	1	0.5%	99.5%	-	-	-
**22th closest**	-	-	-	1	0.5%	100.0%	-	-	-
**23th closest**	-	-	-	0	0.0%	100.0%	-	-	-
**24th closest**	-	-	-	0	0.0%	100.0%	-	-	-

The number (Nº), the percentage (%) and the cumulative percentage (Cum %) of facial features classified in each cluster are shown.

## Discussion

Classification systems to categorize human body parts, or taxonomies obtained from them, provide a standardized way to describe or configure the human body, and a lot of work has been done to categorize many different body parts. Describing facial features using a common terminology is essential in disciplines such us ergonomics, forensics, surgery or criminology. Moreover, the growth of new technologies that use virtual interlocutors or avatars has led to an increasing interest in synthetizing faces and facial expressions that symbolize the user’s presence in new human-machine interaction systems and online activities.

However, there are very few classification systems or taxonomies for facial features, probably due to the complexity of this task, and to limited human capacity for processing individual features compared to the capacity for processing whole faces. Classifying the appearance of facial features requires a holistic approach that considers all visible information. Therefore, encoding the geometry and carrying out a metric or morphological assessment is not enough to obtain facial features taxonomies based on appearance. In this work, appearance-based representations (Eigenfaces) are used to classify the facial features. The developed procedure forms groups of features taking into account all available information and encompassing their global nature.

This procedure was used to classify the facial features of 290 images of males with neutral expression from the Chicago Face Database, obtaining taxonomies of eyes, mouths, and noses for several ethnic groups. To validate the procedure, the agreement of human evaluators with the proposed taxonomies was measured. Out of 200 cases for each feature, 41.0% of mouths, 31.0% of eyes and 46.5% of noses, were classified by humans in the same cluster as in the automatic procedure. More than 73.0% of the features were classified in the expected cluster or in the three clusters closest to it (75.5% of mouths, 73.0% of eyes and 81.0% of noses).

To the best of our knowledge, there are no similar studies to compare these results. In [12], the applicability and feasibility of the DMV atlas [43] was tested measuring the inter-observer and intra-observer errors when classifying several morphological features of male faces (e.g. head shape, nose bridge length, chin shape…). As an example, in this test the shape of the chin was classified into three classes. Despite the low number of classes, the inter-observer error was approximately 39%, while the intra-observer error was 30% for inexperienced observers. These results reflect the subjectivity and the wide variability when judging facial features; every observer showed a specific recognition pattern for the individual facial features. Moreover, this study also concluded that the morphologic assessment of faces is affected by cultural variables. Although more tests must be carried out, in the light of these results it can be concluded that the proposed automatic procedure is a good approach to classify facial features.

Nevertheless, this study has some limitations. The experiment carried out employed 290 images of males with neutral expression from the Chicago Face Database. Therefore, the taxonomies obtained are only representative of the features of the faces belonging to this database. The representativeness of these taxonomies with respect to other populations must be carefully analyzed before their use. The objective of this work was not to obtain the taxonomies but to develop the automatic procedure to classify facial features based on their appearance. A more comprehensive face database can be used to obtain more representative taxonomies. Therefore, our future work will be focused on increasing the sample size of faces used to develop the taxonomies. At the same time, we will test the performance of the proposed system when classifying new faces not used to develop the taxonomies, comparing the results with the classification of human observers.

In the same way, the validation of the proposed procedure was performed for the White facial features. The results obtained for Latino, Asian and Black facial features must be tested, and future work must be done to extend this procedure to other facial features like eyebrows, chins or hair, and to obtain taxonomies of facial features from faces of females.

## Conclusions

Although judging the similarity of facial features is a subjective process with wide inter-observer and intra-observer variability, the results of the validation survey developed in this work show that the proposed procedure can be considered appropriate for the automatic classification of facial features based on their appearance. This procedure deals with the difficulties associated to classify features using judgements from human observers, and facilitates the development of facial features taxonomies.
